# Evaluation of the reliability, usability, and applicability of AMSTAR, AMSTAR 2, and ROBIS: protocol for a descriptive analytic study

**DOI:** 10.1186/s13643-018-0746-1

**Published:** 2018-06-13

**Authors:** Allison Gates, Michelle Gates, Gonçalo Duarte, Maria Cary, Monika Becker, Barbara Prediger, Ben Vandermeer, Ricardo M. Fernandes, Dawid Pieper, Lisa Hartling

**Affiliations:** 1 Alberta Research Centre for Health Evidence, Department of Pediatrics, University of Alberta, 11405-87 Avenue NW, Edmonton, Alberta T6G 1C9 Canada; 2 Clinical Pharmacology Unit, Instituto de Medicina Molecular, University of Lisbon, Av. Professor Egas Moniz, 1649-028 Lisbon, Portugal; 3 Centre for Health Evaluation & Research (CEFAR), National Association of Pharmacies, Rua Marechal Saldanha, no 1, 1249-069 Lisbon, Portugal; 4 Department für Humanmedizin, Institut für Forschung in der Operativen Medizin, Universität Witten/Herdecke, Witten, Germany; 5 Department of Pediatrics, Santa Maria Hospital, Lisbon, Portugal

**Keywords:** Reliability, Validity, Systematic reviews, Risk of bias, Quality assessment, Overviews of reviews

## Abstract

**Background:**

Systematic reviews (SRs) of randomised controlled trials (RCTs) can provide the best evidence to inform decision-making, but their methodological and reporting quality varies. Tools exist to guide the critical appraisal of quality and risk of bias in SRs, but evaluations of their measurement properties are limited. We will investigate the interrater reliability (IRR), usability, and applicability of A MeaSurement Tool to Assess systematic Reviews (AMSTAR), AMSTAR 2, and Risk Of Bias In Systematic reviews (ROBIS) for SRs in the fields of biomedicine and public health.

**Methods:**

An international team of researchers at three collaborating centres will undertake the study. We will use a random sample of 30 SRs of RCTs investigating therapeutic interventions indexed in MEDLINE in February 2014. Two reviewers at each centre will appraise the quality and risk of bias in each SR using AMSTAR, AMSTAR 2, and ROBIS. We will record the time to complete each assessment and for the two reviewers to reach consensus for each SR. We will extract the descriptive characteristics of each SR, the included studies, participants, interventions, and comparators. We will also extract the direction and strength of the results and conclusions for the primary outcome. We will summarise the descriptive characteristics of the SRs using means and standard deviations, or frequencies and proportions. To test for interrater reliability between reviewers and between the consensus agreements of reviewer pairs, we will use Gwet’s AC_1_ statistic. For comparability to previous evaluations, we will also calculate weighted Cohen’s kappa and Fleiss’ kappa statistics. To estimate usability, we will calculate the mean time to complete the appraisal and to reach consensus for each tool. To inform applications of the tools, we will test for statistical associations between quality scores and risk of bias judgments, and the results and conclusions of the SRs.

**Discussion:**

Appraising the methodological and reporting quality of SRs is necessary to determine the trustworthiness of their conclusions. Which tool may be most reliably applied and how the appraisals should be used is uncertain; the usability of newly developed tools is unknown. This investigation of common (AMSTAR) and newly developed (AMSTAR 2, ROBIS) tools will provide empiric data to inform their application, interpretation, and refinement.

**Electronic supplementary material:**

The online version of this article (10.1186/s13643-018-0746-1) contains supplementary material, which is available to authorized users.

## Background

Systematic reviews (SRs) of randomised controlled trials (RCTs) represent the best available evidence to guide health care and policy decisions [1]. To be of value, SRs must be conducted following rigorous processes and the methods and results must be fully and transparently reported. Guidance documents like the Cochrane Handbook for Systematic Reviews of Interventions [2] and Preferred Reporting Items for Systematic Reviews and Meta-analyses (PRISMA) statement [3] aim to inform the rigorous conduct and reporting of SRs. Despite the public availability of these guidance documents, an evaluation of the conduct and reporting quality of SRs of biomedical research published in 2014 showed that there remains ample room for improvement [4]. With the increased publication of SRs, overviews of reviews (in which SRs are the unit of analysis) [2] are becoming more popular. Beyond contributing to research waste [5], the poor quality of many SRs complicates the process of conducting overviews of reviews [6]. To date, there is no consensus as to whether poor quality SRs should be included or excluded from overviews of reviews [6], or what quality criteria should inform their inclusion.

Because the quality of published SRs varies [4], readers and overview authors must appraise SR evidence with a critical eye, and tools to facilitate the process exist. Although there is no firm guidance on which tool to use [7, 8], AMSTAR (A MeaSurement Tool to Assess systematic Reviews) [9] is most often endorsed among methods groups [8]. The 11-item tool was developed in 2007 by combining two existing measures [10, 11] and refining the included items following pilot testing and expert input [9]. Designed to be a living document that could be updated along with advances in empirical evidence [9], AMSTAR 2 was recently developed and published [12]. The new 16-item tool allows for the appraisal of SRs that contain both RCTs and non-RCTs. As opposed to AMSTAR, detailed guidance for reviewers is provided.

The methodological quality and risk of bias of SRs are overlapping but distinct concepts. Generally, the methodological quality of SRs refers to the extent to which they have been performed to the highest possible conduct and reporting standards (e.g. Cochrane standards and PRISMA reporting guidance) [2]. By contrast, the risk of bias in SRs refers to the extent to which their results should be believed, sometimes also termed ‘internal validity’ [2, 13]. Although it may be assumed that poor-quality SRs are at high risk of bias and vice versa, such is not always the case [2]. This is because some markers of quality, e.g. whether the authors provided a list of excluded studies, may affect the extent to which a reader can interpret the results, but will not directly result in bias. Moreover, even well-conducted SRs may present evidence that is at high risk of bias (e.g. due to publication bias that was appropriately measured and reported, or only identifying relevant studies that are at high risk of bias). Although AMSTAR and AMSTAR 2 facilitate the broad appraisal of methodological quality, until recently, no tool existed to guide the appraisal of risk of bias within SRs. The newly introduced ROBIS (Risk Of Bias In Systematic reviews) tool fills this gap. Similar to the AMSTAR tools, ROBIS was developed by reviewing existing tools and literature, then refined via a face-to-face meeting and Delphi process with a panel of experts [14].

Owing to the methods for their development, AMSTAR, AMSTAR 2, and ROBIS exhibit good face and content validity [9, 12, 14]. With respect to interrater reliability (IRR), Pieper et al. reviewed studies that examined AMSTAR’s measurement properties and found substantial reliability for most items [15]. The developers of AMSTAR 2 reported moderate or better reliability for most items on a sample of 20 SRs of health care interventions [12]. Bühn et al. [16] and Perry et al. [17] both reported that ROBIS had fair reliability for 16 SRs of occupational health and 15 SRs of fibromyalgia, respectively. With respect to usability, reports indicate that AMSTAR takes 10 to 20 min to administer [12, 15, 18] and AMSTAR 2 takes 15 to 32 min [12]. Recent evaluations have shown that the time to administer ROBIS is substantially longer than for AMSTAR [16, 19]. With respect to whether these tools may be applied to inform the inclusion of SRs in overviews of reviews, Pollock et al. found no statistical association between AMSTAR scores and the direction of the results or direction and strength of the conclusions of SRs of health care interventions [6]. These findings suggest that SRs with lower scores may be excluded from overviews without introducing bias [6]. We are unaware of similar evaluations related to the application of AMSTAR 2 and ROBIS.

Especially for the newer AMSTAR 2 and ROBIS, there is a need for empiric evidence from diverse samples of SRs to inform how they would be best applied and interpreted. Moreover, there is a need for data to help readers understand the similarities and differences between the IRR, usability, and applicability of AMSTAR and AMSTAR 2. For a heterogeneous sample of SRs of therapeutic interventions from the fields of biomedicine and public health, we will assess for each of AMSTAR, AMSTAR 2, and ROBIS (1) the IRR for individual reviewer pairs, and for pairs of reviewers at three international evidence synthesis centres; (2) their usability, based on the time to complete the appraisals and reach consensus; and (3) their applicability, i.e. whether their findings may be applied to inform the inclusion of SRs in overviews, based on associations between the appraisals and the results and conclusions of the SRs.

## Methods

### Collaborating centres

This descriptive analytical study will be undertaken by an international team of investigators with expertise in SR methodology based at three collaborating centres: the Alberta Research Centre for Health Evidence, University of Alberta, Canada (AG, MG, BV, LH); Instituto de Medicina Molecular, University of Lisbon, Portugal (GD, MC, RMF); and Institut für Forschung in der Operativen Medizin, Universität Witten/Herdecke, Germany (MB, BP, DP). The Canadian site will serve as the coordinating centre for the study. We will undertake the study following a protocol decided a priori, as follows. As this protocol does not describe a SR, we have not registered it on PROSPERO. We will report any amendments to the protocol that occur while undertaking the study within the final manuscript, which we will submit for publication in an academic journal.

### Sample selection

To maximise efficiency, we will exploit a previously identified random sample of 300 SRs of biomedical and public health research indexed in MEDLINE in February 2014 [4]. In a descriptive study published in 2016, Page et al. [4] used the same sample to investigate the epidemiologic and reporting characteristics of SRs in these disciplines. The sample was representative of records indexed in the 3 months prior to and following the month of February [4]. Included SRs were all those that met the PRISMA-P (PRISMA for protocols) definition of a SR [20, 21], irrespective of research question, methodological or reporting quality, or included study designs [4]. Records of the following types were excluded: narrative/non-systematic reviews, non-systematic reviews with meta-analysis or meta-synthesis, reviews that used accelerated SR methods, overviews of reviews, scoping reviews, methodological studies that included a systematic search, and protocols or summaries of SRs [4]. Only English-language records were considered [4]. Because we have no external funding for this work, making use of this previously identified sample will substantially reduce the time and resources required to run a search and screen for relevant records.

From the sample identified by Page et al. [4], we will extract the 147 SRs of therapeutic interventions and transfer these to a Microsoft Office Excel (v. 2016, Microsoft Corporation, Redmond, WA) workbook, allocating each record to one row. In an adjacent column (i.e. column 2), we will assign each row a number using Excel’s random number generator (the RAND function). We will then sort the rows by number (i.e. column 2) in ascending order to achieve a randomised list. We will retrieve the full texts of the first 30 SRs of RCTs from this list, which will serve as our test sample. To supplement the information in the SRs, we will make use of a priori published protocols. If not referenced in the SR, we will search PROSPERO (https://www.crd.york.ac.uk/prospero/) and Google.ca using the titles, authors, and keywords to identify relevant protocols.

### Data collection

All data for this study will be collected following a data collection guide designed by the research team and stored in an Excel workbook. Unless otherwise specified, all descriptive data will be extracted by one reviewer and verified by another reviewer to identify and correct errors.

#### Characteristics of the sample

For each included SR, we will extract characteristics of the publication (authors, year, journal, Cochrane or non-Cochrane), included studies (number, design), participants (number, age, gender, condition), intervention(s), and comparator(s). To test for applicability, we will extract the results and conclusions for the primary outcome of each SR. If multiple primary outcomes are reported, we will consider the first one listed in the Methods of the report to be the primary outcome. When not reported explicitly, we will use a series of decision rules to decide the primary outcome [22, 23]. First, we will consider the outcome named in the title or objective(s) to be the primary outcome. When it is not clear from the title or objective(s), we will consider the most serious outcome (e.g. mortality) to be the primary outcome. To determine the results and conclusions for the primary outcome when multiple interventions are tested, we will use the findings from the comparison of the experimental intervention to placebo or usual care. If it is not clear which one of the interventions is the experimental intervention, we will use the first intervention listed in the results section. Following published criteria [6, 22–25] (Table 1), we will classify the results as favourable, neutral, or unfavourable, and the authors’ conclusions as positive-strong, positive-weak, neutral, negative-weak, or negative-strong. We expect that some SRs will include only narrative syntheses, which we have accounted for in our criteria for classifying results and conclusions. Because authors are not likely to use standard phrasing to describe their findings, two independent reviewers will extract data (numeric or text snippets) and reach consensus regarding the results and conclusions of the SRs.

**Table 1:** Classification scheme for results and conclusions related to the primary outcome [6, 22–25]

Classification	Observation
Results	
Favourable	*P* < 0.10 in favour of the intervention, or described as ‘statistically significant’
Neutral	*P* > 0.10, or described as ‘no difference between groups’
Unfavourable	*P* < 0.10 in favour of the comparator, or described as ‘favouring the non-intervention comparator’
Conclusions	
Positive-strong	Authors state that there is clear evidence for the effectiveness of the intervention, and no further research is needed
Positive-weak	Authors state that there is evidence for the effectiveness of the intervention, but more research is needed to confirm the findings
Neutral	Authors state that there is no or insufficient evidence about whether the intervention is effective or not, and more research is needed to reach a conclusion
Negative-weak	Authors state that there is evidence against the use of the intervention, but more research is needed to confirm the findings
Negative-strong	Authors state that there is clear evidence against the use of the intervention, and no further research is needed

#### Training and pilot testing

Before starting the reliability and usability testing, the two reviewers at each centre (*n* = 6 reviewers) (AG, MG, GD, MC, MB, BP) and three method experts (LH, RF, DP) will independently familiarise themselves with the three tools by reviewing the following documents: the AMSTAR tool, including brief guidance for each item available in Appendix A of Shea et al.’s study of AMSTAR’s reliability and validity [18]; the AMSTAR 2 tool and guidance document available as Additional file 1 to the report published in *The BMJ* in September 2017 [12]; and the ROBIS tool and guidance document available at http://www.bristol.ac.uk/population-health-sciences/projects/robis/.

After becoming familiar with the tools, the reviewers and methods experts will independently pilot test each tool on four SRs. From the previously described randomised list, we will retrieve the full texts and search for protocols for the first four records that meet the following criteria: (1) a Cochrane SR with meta-analysis, (2) a Cochrane SR without meta-analysis, (3) a non-Cochrane SR with meta-analysis, and (4) a non-Cochrane SR without meta-analysis. Following independent appraisal, the review team (reviewers and methods expert) at each centre will convene to discuss inconsistencies in interpretations of the items for each tool. If there are serious differences in the application of the tools in the pilot round, additional pilot testing will be undertaken. Independently at each centre, the review teams will decide on internal decision rules to facilitate the use of each tool, if necessary.

Our reviewers are not experienced in estimating usability via measuring the time to completion of quality and risk of bias appraisals, or for reaching consensus. For this reason, the reviewers and methods experts will independently practice timing their appraisals during the pilot round using a digital chronograph. The time to complete each tool will start when the reviewer begins reading the SR and applying the tool (which may occur simultaneously) and will end when the appraisal is fully complete. The time to reach consensus for each tool and each SR will start once the reviewers convene and will end when agreement is established. Any issues or inconsistencies in measurement will be discussed by all reviewers and methods experts (from all centres), who will decide upon a standardised measurement process before moving forward with formal data collection.

#### Quality and risk of bias appraisals

After the pilot phase, the reviewers will independently apply the AMSTAR, AMSTAR 2, and ROBIS tools for each SR in the test sample. Additional file 1 shows the details of the items and response options for each tool [9, 12, 14]. For the AMSTAR tool, the reviewers will apply a decision of yes, no, cannot answer, or not applicable to each of the 11 items. For the AMSTAR 2 tool, the reviewers will apply a decision of yes or no on items 1, 3, 5, 6, and 10 through 16, and yes, partial yes, or no on items 2, 4, 7, 8, and 9. For items 11, 12, and 15, the reviewers may also choose a decision of not applicable. Based on the decision rules suggested by Shea et al. for AMSTAR 2, the reviewers will apply a rating of high, moderate, low, or critically low for the overall confidence in the results of the review [12]. For the ROBIS tool, the reviewers will apply a decision of yes, probably yes, probably no, no, or no information to each of the signalling questions within the four risk of bias domains and overall. The reviewers will apply a risk of bias rating of low, high, or unclear to each domain and overall. For each SR in the list, the reviewers will apply all three tools before moving to the next. Once complete, the two reviewers at each centre will convene and reach consensus. If the reviewers cannot reach consensus, the methods expert at their centre will adjudicate.

#### Usability assessment

To test usability, we will record the time taken to complete each tool for each SR and for the two reviewers to reach consensus using a digital chronograph, to the nearest second. We will use the standardised process for measuring time to completion and to reach consensus, as decided in the pilot round. Because the reviewers will be familiar with the SRs after one appraisal is complete, and the tools contain similar items, we expect that the second and third tools applied in a series may be completed more efficiently compared to if they had been applied in isolation. For this reason, we will randomise the sequence of assessments such that each reviewer applies either AMSTAR, AMSTAR 2, or ROBIS first within the series for one third of the SRs. We will also collect time data for the tools applied second or third. We will also randomise the order in which the consensus decisions are undertaken, such that for one third of all reviews, agreement will be reached for either AMSTAR, AMSTAR 2, or ROBIS first in a series. We will also collect time to reach consensus for the tools investigated second or third.

### Data analysis

We will transfer all data from the Excel workbook to SPSS Statistics (v*.* 24, International Business Machines (IBM) Corporation, Armonk, NY) or StatXact (v*.* 11, Cytel, Cambridge, MA) for analysis. We will recode the textual data extracted from the studies and from the quality and risk of bias assessments into numeric categories as appropriate. We will summarise the characteristics of the sample of SRs individually in a table. To characterise the sample as a whole, we will use descriptive statistics, including frequencies and proportions for categorical data and means and standard deviations (SDs) for continuous data. Although not recommended in practice [9, 12], for the purposes of this study, we will calculate an overall AMSTAR quality score by summing the number of ‘yes’ responses and dividing these by the total number of items for each tool. We will subtract the ‘not applicable’ items from the total number of items (denominator) for this calculation. Similar methods were used by AMSTAR’s developers to validate and test the IRR of the tool [9, 18] and will allow for comparability to previous evaluations.

For each item and overall for each tool, we will calculate reliability between reviewers and the consensus of reviewer pairs between centres using the Gwet’s AC_1_ statistic [26], with 95% confidence intervals (CIs). To date, most evaluations of AMSTAR and ROBIS have used the Kappa statistic to measure agreement [15–17], but when sample sizes are small and agreement between reviewers is high, Kappa may underestimate true IRR [26, 27]. To maintain comparability to previous evaluations, in addition to Gwet’s AC_1_, we will use the weighted Cohen’s kappa statistic as described by Liebetrau [28] to determine IRR, and Fleiss’ kappa statistic [29] to determine reliability between the consensus of reviewer pairs. Agreement based on both the AC_1_ and Kappa statistics will be interpreted following the recommendations of Landis and Koch [30] as follows: poor (< 0), slight (0.0–2.0), fair (0.21–0.40), moderate (0.41–0.60), substantial (0.61–0.80), or almost perfect (0.81–1.0). To estimate usability, we will calculate the mean time (SD) to completion for each tool, and to the completion of the consensus decisions. To determine applicability, for each tool, we will test for statistical associations between the results and conclusions for the primary outcome of each SR and overall score, per site.

The absence of specific statistical tests or hypotheses to be tested precludes sample size calculations. Similar studies, however, have successfully used similar sample sizes to that which we have proposed. For example, Banzi et al. used a sample of 31 SRs of thromboprophylaxis to test the IRR and usability of AMSTAR and ROBIS [19]; Harting et al. used a sample of 30 RCTs to test the IRR of the Cochrane Risk of Bias tool [31]; Shea et al. used a sample of 42 SRs to inform the external validation of AMSTAR [18]; Kang et al. used a sample of 41 SRs of Chinese traditional medicine to test the IRR of AMSTAR [32]; and Pieper et al. used a sample of 16 SRs of occupational health to test the IRR of AMSTAR between various reviewer pairs [33]. The sample size was thus informed by previous work and decided following a pragmatic approach considering the availability of resources and personnel. The precision with which we will be able to estimate the values of AC_1_ and Kappa will depend upon the nature of the agreement between reviewers in each of the components. For Cohen’s kappa, we expect estimates with standard errors between about 0.04 and 0.17. For Gwet’s AC_1_, we expect estimates with standard errors between 0.10 and 0.17.

## Discussion

The methodological and reporting quality of SRs can vary [4], and when their conduct is poor, the results can be biased. Which tool provides the most reliable and valid assessment of SR quality, and the usability of newly available tools, is not known. Descriptive analyses of overviews of reviews [34–36] suggest that authors do not universally undertake risk of bias and/or quality assessments of SRs before drawing conclusions. Barriers to the use of available tools may include the real, or perceived time and resources necessary to complete them, and reviewers’ confidence in their own appraisals. Our study will provide empirical data on the reliability, usability, and applicability of three tools that have undergone rigorous development processes [9, 12, 14]. The findings may inform their application, interpretation, and refinement.

### Strengths and limitations

To our knowledge, this will be one of few studies that have tested and compared the reliability, usability, and applicability of AMSTAR, AMSTAR 2, and ROBIS [16, 17]. Our study is strengthened by the fact that we will use a random sample of SRs that is heterogeneous with respect to size (number of participants and studies included) and quality of conduct and reporting [4]. Reviewers from various backgrounds and with different levels of experience will test the tools, mimicking real-world conditions where individuals with a range of expertise are involved in quality and risk of bias appraisal. The planned self-directed training, pilot round, and development of decision rules at each centre will likely improve IRR. Although standard guidance for undertaking overviews of reviews does not exist [8], Pollock et al. recommended the development of internal decision rules to improve IRR for author groups who apply AMSTAR [6]. Because we cannot ascertain whether our methods of training and piloting are universal to most centres, we cannot ensure that the findings will be generalisable. Due to time and resource constraints, we will limit our sample to 30 SRs of therapeutic interventions including only RCTs, which could compromise generalisability and precision.

### Dissemination

The findings of this study will be of interest to clinicians and policymakers who rely on SRs and overviews of reviews to guide clinical practice and policy decisions. They will also be of interest to authors and readers of SRs and overviews of reviews, who ideally would use the tools in their work. Using a multi-modal dissemination strategy, including the publication of our results in an academic journal, presentations at multidisciplinary conferences, and social media messages, we will ensure an adequate reach.

## Additional file

Additional file 1: Items and response options on the AMSTAR, AMSTAR 2, and ROBIS tools. Provides an overview of the items and response options on the AMSTAR, AMSTAR 2, and ROBIS tools. (DOCX 20 kb)

## Abbreviations

AMSTAR: A MeaSurement Tool to Assess systematic Reviews
CI: Confidence interval
IRR: Interrater reliability
PRISMA: Preferred Reporting Items for Systematic reviews and Meta-Analyses
RCT: Randomised controlled trial
ROBIS: Risk Of Bias In Systematic reviews
SD: Standard deviation
SR: Systematic review
