# Supplementary material for: Evaluation of the reliability, usability, and applicability of AMSTAR, AMSTAR 2, and ROBIS: protocol for a descriptive analytic study
Source: Syst Rev. 2018 Jun 13;7:85. doi: 10.1186/s13643-018-0746-1 (PMC6000957; doi:10.1186/s13643-018-0746-1)
Supplement: Supplementary file 1 — Items and response options on the AMSTAR, AMSTAR 2, and ROBIS tools. Provides an overview of the items and response options on the AMSTAR, AMSTAR 2, and ROBIS tools. (DOCX 20 kb) [file 13643_2018_746_MOESM1_ESM.docx]

**Additional File 1.** Items and response options on the AMSTAR, AMSTAR 2, and ROBIS tools

| **Items** | **Response Options** |
| --- | --- |
| **AMSTAR** [9] | |
| 1. Was an ‘a priori’ design provided? | Yes; No; Can’t Answer; Not applicable |
| 1. Was there duplicate study selection and data extraction? |  |
| 1. Was a comprehensive literature search performed? |  |
| 1. Was the status of publication used as an inclusion criterion? |  |
| 1. Was a list of studies (included and excluded) provided? |  |
| 1. Were the characteristics of the included studies provided? |  |
| 1. Was the scientific quality of the included studies assessed and documented? |  |
| 1. Was the scientific quality of the included studies used appropriately in formulating conclusions? |  |
| 1. Were the methods used to combine the findings of studies appropriate? |  |
| 1. Was the likelihood of publication bias assessed? |  |
| 1. Was the conflict of interest included? |  |
| **AMSTAR 2** [12] | |
| 1. Did the research questions and inclusion criteria for the review include the components of PICO? | Yes; No |
| 1. Did the report of the review contain an explicit statement that the review methods were established prior to conduct of the review and did the report justify any significant deviations from the protocol? | Yes; Partial yes; No |
| 1. Did the review authors explain their selection of the study designs for inclusion in the review? | Yes; No |
| 1. Did the review authors use a comprehensive literature search strategy? | Yes; Partial yes; No |
| 1. Did the review authors perform study selection in duplicate? | Yes; No |
| 1. Did the review authors perform data extraction in duplicate? | Yes; No |
| 1. Did the review authors provide a list of excluded studies and justify the exclusions? | Yes; Partial yes; No |
| 1. Did the review authors describe the included studies in adequate detail? | Yes; Partial yes; No |
| 1. Did the review authors use a satisfactory technique for assessing the risk of bias in individual studies that were included in the review? | (a) RCTs: Yes; Partial yes; No; Includes only NRSI (not applicable)  (b) NRSIs: Yes; Partial yes; No; Includes only RCTs (not applicable) |
| 1. Did the review authors report on the sources of funding for the included studies? | Yes; No |
| 1. If meta-analysis was performed, did the review authors use appropriate methods for statistical combination of results? | (a) RCTs: Yes; No; No meta-analysis conducted  (b) NRSI: Yes; No; No meta-analysis conducted |
| 1. If meta-analysis was performed, did the review authors assess the potential impact of risk of bias in individual studies on the results of the meta-analysis of other evidence synthesis? | Yes; No; No meta-analysis conducted |
| 1. Did the review authors account for risk of bias in individual studies when interpreting/discussing the results of the review? | Yes; No |
| 1. Did the review authors provide a satisfactory explanation for, and discussion of, any heterogeneity observed in the results of the review? | Yes; No |
| 1. If they performed quantitative synthesis did the review authors carry out an adequate investigation of publication bias (small study bias) and discuss its likely impact on the results of the review? | Yes; No; No meta-analysis conducted |
| 1. Did the review authors report any potential sources of conflict of interest, including any funding they received for conducting the review? | Yes; No |
| Overall confidence in the results of the review | High, Moderate, Low, Critically Low |
| **ROBIS** [14] | |
| 1. Domain 1: Study eligibility criteria | |
| 1. Did the review adhere to pre-defined objectives and eligibility criteria? | Yes; Probably yes; Probably no; No; No information |
| 1. Were the eligibility criteria appropriate for the review question? |  |
| 1. Were eligibility criteria unambiguous? |  |
| 1. Were all restrictions in eligibility criteria based on study characteristics appropriate? |  |
| 1. Were any restrictions in eligibility criteria based on sources of information appropriate? |  |
| Concerns regarding specification of study eligibility criteria | Low; High; Unclear |
| 1. Domain 2: Identification and selection of studies | |
| 1. Did the search include an appropriate range of databases/electronic sources for published and unpublished reports? | Yes; Probably yes; Probably no; No; No information |
| 1. Were methods additional to database searching used to identify relevant reports? |  |
| 1. Were the terms and structure of the search strategy likely to retrieve as many eligible studies as possible? |  |
| 1. Were restrictions based on date, publication format, or language appropriate? |  |
| 1. Were efforts made to minimise error in selection of studies? |  |
| Concerns regarding methods used to identify and/or select studies | Low; High; Unclear |
| 1. Domain 3: Data collection and study appraisal | |
| 1. Were efforts made to minimise error in data collection? | Yes; Probably yes; Probably no; No; No information |
| 1. Were sufficient study characteristics available for both review authors and readers to be able to interpret the results? |  |
| 1. Were all relevant study results collected for use in the synthesis? |  |
| 1. Was risk of bias (or methodological quality) formally assessed using appropriate criteria? |  |
| 1. Were efforts made to minimise error in risk of bias assessment? |  |
| Concerns regarding methods used to collect data and appraise studies | Low; High; Unclear |
| 1. Domain 4: Synthesis and findings | |
| 1. Did the synthesis include all studies that is should? | Yes; Probably yes; Probably no; No; No information |
| 1. Were all pre-defined analyses reported or departures explained? |  |
| 1. Was the synthesis appropriate given the nature and similarity in the research questions, study designs and outcomes across included studies? |  |
| 1. Was between-studies variation (heterogeneity) minimal or addressed in the synthesis? |  |
| 1. Were the findings robust, e.g., as demonstrated through funnel plot or sensitivity analyses? |  |
| 1. Were biases in primary studies minimal or addressed in the synthesis? |  |
| Concerns regarding the synthesis and findings | Low; High; Unclear |
| 1. Risk of bias in the review (overall) | |
| 1. Did the interpretation of findings address all of the concerns identified in Domains 1 to 4? | Yes; Probably yes; Probably no; No; No information |
| 1. Was the relevance of identified studies to the review’s research question appropriately considered? |  |
| 1. Did the reviewers avoid emphasizing results on the basis of their statistical significance? |  |
| Risk of bias in the review | Low; High; Unclear |
